# Virtual Non-Contrast Spectral CT in Renal Masses: Is It Time to Discard Conventional Unenhanced Phase?

**DOI:** 10.3390/jcm12144718

**Published:** 2023-07-17

**Authors:** Giuseppe M. Bucolo, Velio Ascenti, Simone Barbera, Federico Fontana, Francesco M. Aricò, Filippo Piacentino, Andrea Coppola, Giuseppe Cicero, Maria Adele Marino, Christian Booz, Thomas J. Vogl, Tommaso D’Angelo, Massimo Venturini, Giorgio Ascenti

**Affiliations:** 1 Department of Biomedical Sciences and Morphological and Functional Imaging, University of Messina, 98122 Messina, Italy; 2 Postgraduation School in Radiodiagnostics, Università degli Studi di Milano, Via Festa del Perdono 7, 20122 Milan, Italy; 3 Diagnostic and Interventional Radiology Unit, ASST Settelaghi, Insubria University, 21100 Varese, Italy; 4 Division of Experimental Imaging, Department of Diagnostic and Interventional Radiology, University Hospital Frankfurt, 60590 Frankfurt am Main, Germany; 5 Department of Radiology and Nuclear Medicine, Erasmus MC, 3015 GD Rotterdam, The Netherlands

**Keywords:** virtual non-contrast image, dual energy, computed tomography, findings incidental kidney

## Abstract

Dual-layer Dual-Energy CT (dl-DECT) allows one to create virtual non-contrast (VNC) reconstructions from contrast-enhanced CT scans, with a consequent decrease of the radiation dose. This study aims to assess the reliability of VNC for the diagnostic evaluation of renal masses in comparison with true non-contrast (TNC) images. The study cohort included 100 renal masses in 40 patients who underwent dl-DECT between June and December 2021. Attenuation values and standard deviations were assessed through the drawing of regions of interest on TNC and VNC images reconstructed from corticomedullary and nephrographic phases. A Wilcoxon signed-rank test was performed in order to assess equivalence of data and Spearman’s Rho correlation coefficient to evaluate correlations between each parameter. The diagnostic accuracy of VNC was estimated through the performance of receiver operating characteristic (ROC) curve analysis. Differences between attenuation values were, respectively, 74%, 18%, 5% and 3% (TNC-VNC_cort_), and 74%, 15%, 9% and 2% (TNC-VNC_neph_). The Wilcoxon signed-rank test demonstrated the equivalence of attenuation values between the TNC and VNC images. The diagnostic performance of VNC images in the depiction of kidney simple cysts remains high compared to TNC (VNC_cort_-AUC: 0.896; VNC_neph_-AUC: 0.901, TNC-AUC: 0.903). In conclusion, quantitative analysis of attenuation values showed a strong agreement between VNC and TNC images in the evaluation of renal masses.

## 1. Introduction

As is well known, the unquestionable advantages of CT-scan, such as a high spatial and contrast resolution as well as rapid scan times, are counterbalanced by potential health risks related to radiation exposure [1,2,3,4].

In order to overcome this critical issue, over the last decades, specific reconstruction algorithms, for instance iterative or deep-learning-based ones, have been developed [5,6,7,8].

However, it should be kept in mind that radiation dose can be easily decreased by just avoiding unnecessary scans when multiphase CT protocols are used.

Dual-energy computed tomography (DECT) is an already established imaging technology that exploits images’ raw data obtained at different energy levels in order to achieve material decomposition [9,10].

This concept includes the differentiation of body-tissue components according to the atomic number, with the possibility of highlighting or minimizing their effect on the final image using dedicated post-processing algorithms.

Among these, virtual non-contrast (VNC) aims at removing iodine from contrast-enhanced images [11]. Since iodine is the main element of intravenous contrast medium, the result is an unenhanced set of images obtained without the need for a real acquisition. The consequent advantages in terms of the spareness of radiation dose have been made evident.

In the abdominal field, the use of VNC has shown encouraging results, particularly in the characterization of hepatic and pancreatic lesions [12,13].

Conventional non-contrast imaging plays a crucial role in the comprehensive evaluation and characterization of renal masses, providing accurate identification of common lesions with confidence. It provides essential information for classifying cystic kidney masses based on their attenuation values; for instance, a homogeneous mass measuring 70 HU or higher at the non-contrast phase indicates a high probability of representing a high-attenuation renal cyst [14]. Additionally, unenhanced CT is highly effective in detecting calcification or macroscopic fat tissue, contributing to accurate diagnoses. In fact, the presence of macroscopic fat within a solid renal mass allows for differentiation between classical angiomyolipomas and other lesions, such as renal cell carcinoma [15].

Thus, the purpose of this study is to evaluate the reliability of attenuation values of renal masses in VNC images, compared to true non-contrast (TNC) images, using a dual-layer (dl) DECT scanner.

## 2. Materials and Methods

### 2.1. Study Population

A retrospective single-center study was conducted.

All contrast-enhanced abdominal dl-DECT scans performed with a multiphasic scan protocol in patients with a clinical suspicion of renal masses between June 2021 and December 2021, present on our Picture archiving and communication system (PACS), were reviewed. No preselection regarding patients’ weight, age, sex, or other characteristics was made.

**Figure 1:**
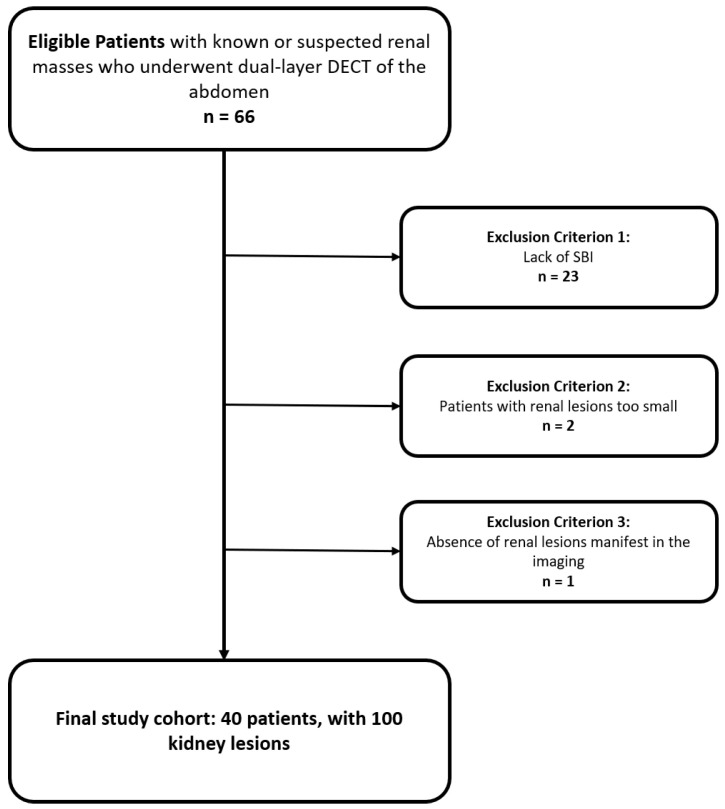
Flowchart of patient inclusion and exclusion criteria.

Only patients with renal lesions larger than 5 mm at a maximum diameter have been included in the final patient cohort (Figure 1).

### 2.2. DECT Scan Protocol

All scans were obtained using a clinical dl-DECT scanner (IQon, Philips Healthcare, Best, the Netherlands). Patients were examined in supine position with a field of scan ranging from the diaphragm to the pubic symphysis. Image acquisition was performed in the craniocaudal direction during inspiratory breath hold.

The study protocol consisted of unenhanced, cortico-medullary and nephrographic phases.

A bolus tracking ROI was positioned on the descending aorta, and the cortico-medullary phase started 15 s after reaching the 100 HU trigger threshold. Nephrograpic phase scans was subsequently acquired at a time delay of 70–80 s after bolus tracking. The settings used for dl-DECT imaging were: tube voltage 120 kVp, tube current modulation activated (DoseRight 3D-DOM, Philips Healthcare), gantry rotation time 0.33 s, pitch 0.9, collimation 64 × 0.625 mm, rotation time 0.5, and volume CT dose index (CTDIvol) of 2.9 milligrays (mGy).

The intravenous contrast agent (Iomeron 400 mgI/mL, Bracco) was injected at a dose of 1.2 mL per kilogram body weight and at a flow rate of 3–4 mL/s and followed by a bolus of 40 mL of saline. No oral contrast material was administered.

Technical parameters are summarized in Table 1.

### 2.3. Image Reconstruction

Axial conventional images with a 2.00 mm slice thickness were reconstructed from contributions of both detector layers using the proprietary iDose4 reconstruction algorithm.

Spectral database images (SBI) were automatically generated in order to obtain post-processing reconstructions through a dedicated dl-DECT WorkStation (IntelliSpace Portal (vv. 8.0.2), Philips Healthcare, Best, Netherlands).

**Figure 2:**
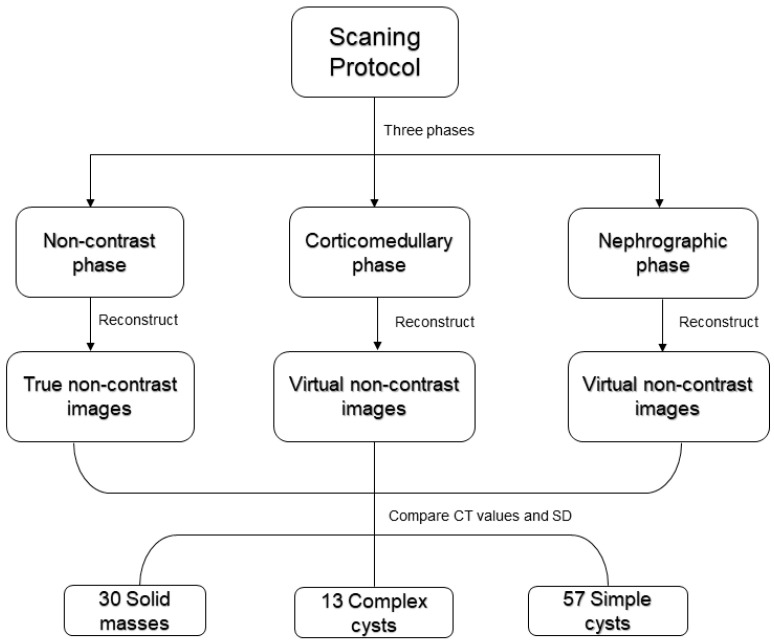
Flowchart of study design.

SBI data were exploited to create VNC image series starting from the cortico-medullary and nephrographic phases (Figure 2).

### 2.4. Objective Image Evaluation

Two radiologists, with 3 and 5 years of experience, respectively, in abdominal radiology, evaluated the renal masses, drawing a circular region of interest (ROI within kidney lesions, covering the largest area at the level of the maximum diameter). The ROIs’ size areas ranged between 17.41–4318.32 mm^2^ and were kept constant across the different image series of the same patient.

Attenuation values measured on TNC images served as the reference standard.

Mean values of measurements were used for the statistical analysis.

### 2.5. Statistical Analysis

Statistical analysis was performed using dedicated software (R version 4.2.0, R Foundation for Statistical Computing, Vienna, Austria; Matlab, Matworks v. R2022a, Natick, MA, USA; Excel 365, Microsoft, Redmond, WA, USA).

The non-normality distribution of data (CT values and SD) was evaluated using both Shapiro–Wilk and Kolmogorov–Smirnov tests. A Wilcoxon signed-rank test was performed using a TOST (two one-sided test) equivalence test to reject the null hypothesis. The null hypothesis argued that the difference between the median of the attenuation values in TNCs and VNCs was greater than 3/5/10 HU. The statistical test was conducted while considering overall lesions and then dividing lesions into three groups (renal masses and cystic lesions, the latter further classified as simple or complex).

The statistically significant difference was indicated by a *p* value under 0.05.

Moreover, Spearman’s Rank correlation coefficient was assessed between CT values and standard deviations (HU) measured on TNC and VNC of the corticomedullary phase (VNC_cort_) and those on TNC and VNC of the nephrographic phase (VNC_neph_).

A Bland–Altmann plot was drawn to assess the agreement of VNC attenuation values in comparison to the standard conventional imaging attenuation values obtained from TNC [16].

The difference between attenuation values on TNC and VNC_cort_ images was calculated while evaluating how many times it was under 5 HU, between 5–10 HU and 10–20 HU, or over 20 HU. The same was performed between TNC and VNC_neph_.

The receiver operating characteristic curves (ROC) and the associated area under the curve (AUC) with a 95% confidence interval were assessed to compare the sensitivity and sensibility of VNC images and unenhanced images. The difference between the curves was evaluated by using the DeLong test for correlated curves.

## 3. Results

### 3.1. Population Characteristics

From a total of 66 patients, the following patients were excluded: 23 (33%) because of the lack of SBI data storage on PACS, 2 (3%) because the lesion’s maximum diameter was smaller than 5 mm, and 1 (1%) due to absence of renal masses.

The final study cohort consisted of 40 patients (30 males, mean age: 68.19 years; 36–87 years; 10 females, mean age: 61.60, range 35–81).

A total of 100 masses were included in the study, consisting of 30 renal solid masses, 13 complex cysts, and 57 simple cysts.

### 3.2. Quantitative Assessment

**Figure 3:**
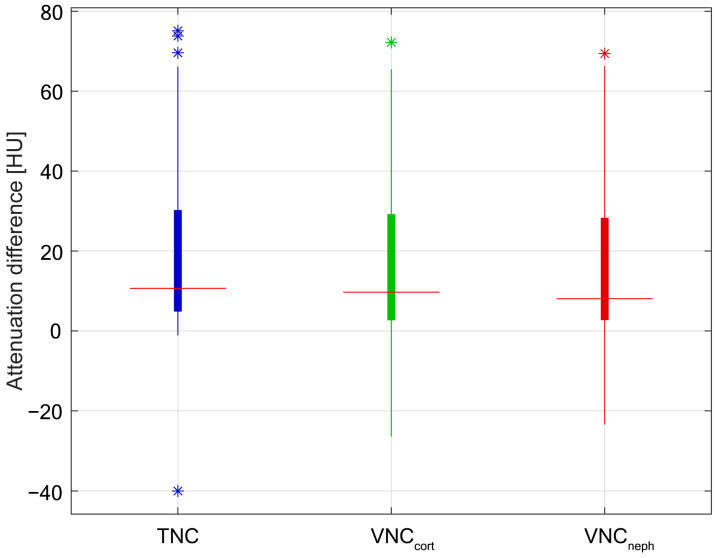
The boxplot shows a similar distribution of TNC and VNC attenuation values. Asterisks (*) represent outlier values. TNC—True non-contrast; VNC_cort_—Virtual non-contrast of corticomedullary phase; VNC_neph_—VNC of nephrographic phase.

The median values of data obtained from TNC, VNC_cort_, and VNC_neph_ are 10.65 HU, 9.70 HU, and 8.05 HU, respectively. The maximum attenuation value is 75.10 HU in TNC images, compared with 72.20 and 69.40 HU on VNC_cort_ and VNC_neph_, respectively; meanwhile, the minimum attenuation value is −40.00 HU on TNC series, −26.40 HU on VNC_cort_ series, and −23.4 HU on VNC_neph_ series (Figure 3).

The means of all CT values of renal masses obtained from TNC and VNC reconstructions are summarized in Table 2.

Overall, the difference in attenuation values on TNC and VNC_cort_ images was lower than 5 HU in 74% of cases, between 5–10 HU in 18% of cases, between 10–20 HU in 5% of cases, and greater than 20 HU in 3% of cases.

**Figure 4:**
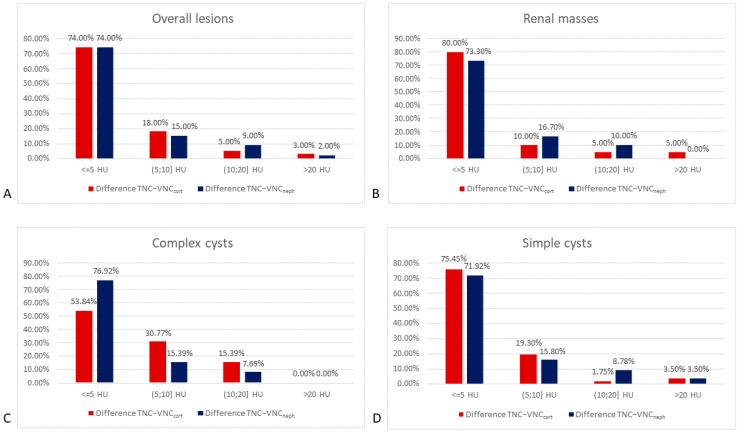
The graph shows how frequently the differences between TNC and VNC attenuation values (HU) are <5, in the range of 5–10, of 10–20, and >20 for (**A**) overall kidney masses, (**B**) kidney masses, (**C**) complex cysts, and (**D**) simple cysts. TNC—True non-contrast; VNC_cort_—VNC of corticomedullary phase; VNC_neph_—VNC of nephrographic phase.

The attenuation values obtained by the TNC and VNC_neph_ differ by 5 HU in 74% of cases, while the difference is between 5–10 HU, between 10–20 HU, and greater than 20 HU, respectively, in 15%, 9%, and 2% of cases (Figure 4A–D).

Considering each group individually (kidney masses, simple and complex cysts), the results obtained are similar to those obtained when evaluating all the lesions.

Spearman’s Rank correlation coefficient (Spearman rho) between TNC and VNC_cort_ was 0.87, while it was 0.89 between TNC and VNC_neph_.

The results of the Wilcoxon signed-rank test based on TOST rejected the null hypothesis, indicating that the differences between all the median values are between the ranges of −3, +3 HU, −5, +5 HU, and −10, +10 HU, so that the attenuation values of TNC and VNC are equivalent (Table 3). The rejection of the null hypothesis has confirmed that the attenuation values obtained from the TNC and VNC image series were equivalent to the threshold assessed.

This is valid in evaluating overall lesions and also each group (kidney masses, simple and complex cysts). Only in the interval −3; +3 does the test not show statistical significance in the depiction of cystic lesions (*p* > 0.05) (Table 3).

**Figure 5:**
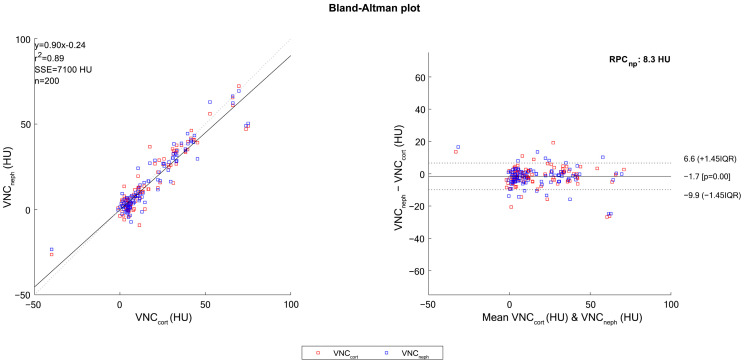
Bland−Altman plot demonstrates the agreement between the measurements using the virtual non-contrast (VNC) attenuation values and unenhanced attenuation values. The horizontal dashed lines represent the mean (1.7 HU) and the limits of the 95% confidence interval (6.6 HU, −9.9 HU) for the difference. VNC_cort_—VNC of corticomedullary phase; VNC_neph_—VNC of nephrographic phase.

The Bland−Altman plot (Figure 5) provides the difference between the attenuation values on single TNC and VNC measurements and their mean values in order to identify the agreement between the measured values in TNC and VNC.

**Figure 6:**
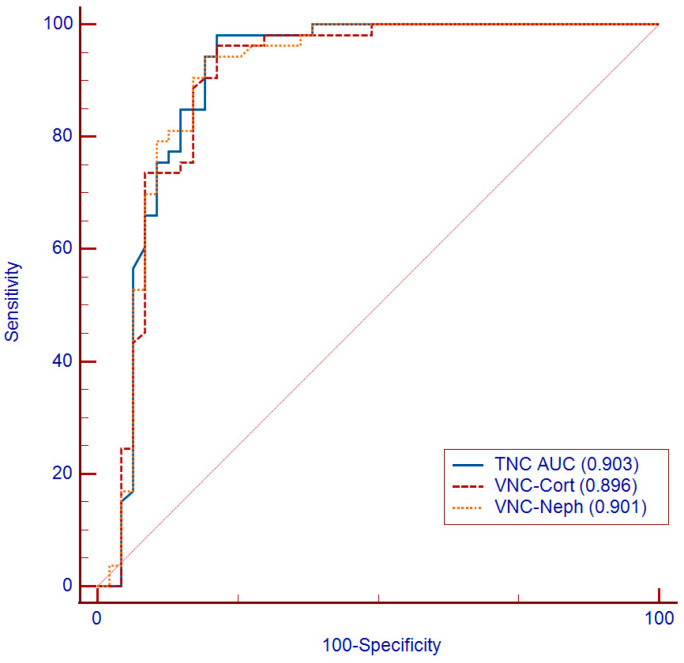
The comparison of ROC curves shows the diagnostic performance of VNC series compared to TNC images. TNC—True non-contrast; AUC—Area under the curve; VNCcort—VNC of corticomedullary phase; VNCneph—VNC of nephrographic phase.

The ROC curves evaluated for simple cystic lesions show a mild difference in diagnostic performance between TNC (AUC: 0.903), considered as a standard reference, VNC_cort_ (AUC: 0.896), and VNC_neph_ (AUC: 0.901). Moreover, the comparison of ROC curves does not show a statistical difference between TNC, VNC corticomedullary, and nephrographic reconstructions (*p*: 0.817 and *p*: 0.944, respectively), confirming the interchangeability of the true and virtual image series (Figure 6).

## 4. Discussion

CT scan plays a key role in the evaluation of renal masses [17]. The main purposes of their characterization are represented by the differentiation of cystic from solid lesions and, within those groups, the distinction of benign from malignant [18].

However, the increasingly widespread use of CT scan over the last decades has raised the issue of radiation dose delivery to the patient, leading radiologists to individualize each examination to the patient in order to minimize potentially harmful effects [19].

In this context, the exploitation of the dl-DECT potential through VNC reconstructions may avoid the performance of a true unenhanced scan and therefore reduce the final radiation exposure.

In this study, the reliability of attenuation values within renal masses obtained on VNC images reconstructed from cortico-medullary and nephrographic phases was assessed and compared to those of TNC.

Our results support the interchangeability of TNC with VNC series. The differences in attenuation values obtained from the VNC_cort_ and TNC series are less than 10 HU in 92% of cases, and only 3% of cases show more than 20 HU. VNC obtained from the nephrographic phase shows attenuation values under 10 HU in 89% of cases but with only 2% of cases having more than 20 HU.

Moreover, the comparison of TNC and VNC attenuation values with the TOST Wilcoxon signed-rank test does not demonstrate a significant difference between the variables (*p* < 0.05), supporting the fact that TNC and VNC images are closely related.

ROC curves showed a high diagnostic accuracy for the detection of kidney simple cysts on the VNC series (VNC_cort_-AUC: 0.896; VNC_neph_-AUC: 0.901; TNC images AUC: 0.903) (Figure 6).

**Figure 7:**
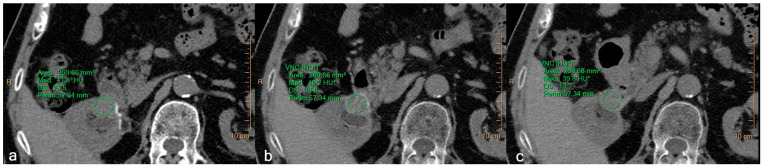
A 78-year-old male patient with recurrent renal cell carcinoma. Equal ROIs were drawn in all three phases: (**a**) TNC, (**b**) VNC obtained from the arterial phase, (**c**) VNC obtained from the venous phase. The attenuation value (HU) and the standard deviations were, respectively, 41.8 ± 13.5, 46.2 ± 16.8, and 39.8 ± 13.7. The difference in attenuation between the real and virtual images is less than 5 HU, and the images are qualitatively comparable, also in terms of the noise level. TNC—True non-contrast; VNC—Virtual non-contrast. Asterisks (*) indicate the HU of VNC images.

In the evaluation of renal cell carcinoma recurrence, the differences in attenuation values and image noise obtained between TNC and VNC reconstructions are essentially equivalent, demonstrating a difference lower than 5 HU (Figure 7).

**Figure 8:**
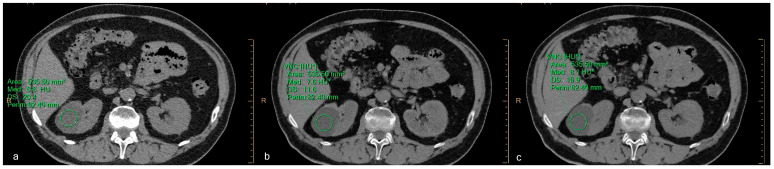
A 71-year-old male patient with a complex cystic lesion (Bosniak type II F). The attenuation values and SD of (**a**) baseline image, (**b**) virtual non-contrast (VNC) obtained from the arterial phase, and (**c**) VNC obtained from the venous phase are, respectively, 6.8 ± 20.4 HU, 7.6 ± 11.6 HU, and 8.1 ± 10.9 HU. The difference between the attenuations is less than 5 HU, and the VNC images have a lower noise level, resulting in a lower SD value. Asterisks (*) indicate the HU of VNC images.

Even for complex cystic lesions (Bosniak II), we found a strong agreement between attenuation values and a slightly lower noise level (Figure 8).

**Figure 9:**
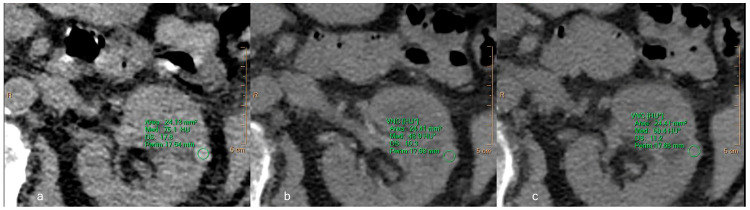
(**a**) Hyperdense cystic lesion in TNC image with an attenuation value of 75.1 HU represents a Bosniak 2 lesion, not to be further investigated. However, in the VNC images obtained from the (**b**) arterial and (**c**) portal-venous phases, the lesion’s attenuation values are, respectively, 48.9 and 50.4 HU. VNC images, in this case, do not allow an accurate and correct diagnosis. TNC—True non-contrast; VNC—Virtual non-contrast. Asterisks (*) indicate the HU of VNC images.

On the contrary, in the evaluation of a native CT hyperdense cystic lesion (Bosniak II), we obtained discordant attenuation values in VNC images, which would have led to an incorrect diagnosis and unnecessary further diagnostic investigations (Figure 9).

Up to now, several in vivo and in vitro studies were performed to assess the reliability of VNC instead of TNC images.

In 2018, Andreas P. Sauter et al. investigated the reliability of VNC in a study with 62 patients. They assessed the attenuation values of TNC and VNC images obtained from the corticomedullary and portal venous phases of different tissues (aorta, fat, muscle, liver kidney, fluid, bone). Their results showed a difference of 1.1 ± 6.7 HU between TNC and VNC for liver, kidney, muscle, and fluid combined tissue, concluding that VNC could represent a promising tool for daily clinical routine [20].

Jasmin A. Holz et al. evaluated the relationship between TNC and dl-DECT VNC using an abdomen phantom with seven different tissue types and compared their attenuation and SD. Their results showed that the VNC error was −1.4 ± 6.1 and independent of dose ranges, kernel, and denoising setting [21].

Meyer et al. demonstrated a close agreement between the attenuation values of real and virtual images. Nonetheless, the mean error rate for the enhancement assessment was 12.1% (8.7% false positives and 3.4% false negatives). Therefore, they concluded that this bias may be accepted in the evaluation of incidental renal masses, but not for CT studies performed to characterize renal masses [22].

Moreover, Zhang et al. evaluated whether VNC images, obtained from the excretory phase, could replace the TNC series in the evaluation of patients with renal cell carcinoma [15]. They found an optimal agreement between VNC and TNC attenuation values, concluding that there was a possible interchangeability of VNC and TNC series.

However, despite the strong agreement between true and virtual non-contrast imaging, we have to keep in mind that VNC reconstructions cannot yet completely replace TNC images, especially when the latter become necessary in differential diagnosis. This could be relevant in cases of cystic lesions, as our study showed statistical significance in the equivalence test within the range of ±5 and ±10 HU, but not within the narrower range of ±3 HU (*p* > 0.05), in both corticomedullary and nephrographic phases.

Nevertheless, in patients who underwent many follow-up imaging examinations throughout their life, especially when young, and in patients from the emergency department with specific clinical questions, VNC reconstructions may be helpful in order to characterize an incidental lesion [20,22], with significant advantages coming from a decrease of radiation exposure [23,24,25].

The limitations of this study must be acknowledged. Firstly, the retrospective study design could have influenced our results. Secondly, we consider only the most frequent renal masses; other kinds of lesions will have to be assessed in future studies because they could show different results. Thirdly, we used just one dual-layer DECT scanner; the validity of VNC reconstructions with other DECT technology may be different, and this has to be evaluated in further studies.

## 5. Conclusions

Our study has demonstrated a strong agreement between VNC and TNC images when evaluating renal lesions. In particular, we observed a difference in attenuation values of less than 10 HU between the VNC_cort_ and TNC series and between the VNC_neph_ and TNC series in 92% and 89% of cases, respectively.

Thus, although the small discrepancies observed in a minority of cases emphasize the diagnostic value of TNC images, especially at the initial characterization of indeterminate renal masses, the VNC algorithm could be reliably exploited in following examinations as a substitute for an unenhanced scan, with relevant benefits for the patient in terms of dose protection.

## Figures and Tables

**Table 1:** Acquisition parameters.

Parameters	DECT
Collimation (mm)	64 × 0.625
Tube voltage (kv)	120
Rotation time (s)	0.33
Tube current (ma)	89
Pitch	0.9
Duration of tube current (s)	0.5
CTDI (mgy)	2.9

DECT—Dual-Energy CT; CTDI—Computed tomography dose index.

**Table 2:** The means of all CT values of renal masses obtained from TNC and VNC reconstructions.

Type of Lesion	Phase	Mean Value (HU)	SD
All lesions	TNC	17.6	16.5
	VNC_cort_	15.7	13.2
	VNC_neph_	15.6	12.7
Simple Cysts	TNC	10.7	15.6
	VNC_cort_	7.8	12.2
	VNC_neph_	7.5	11.9
Complex Cysts	TNC	15.3	18.6
	VNC_cort_	12.7	13.6
	VNC_neph_	13.1	14.4
Renal Masses	TNC	34.3	16.9
	VNC_cort_	34.1	15.1
	VNC_neph_	33.9	13.4

TNC—True non-contrast; VNC_cort_—Virtual non-contrast of corticomedullary phase; VNC_neph_—VNC of nephrographic phase.

**Table 3:** Results of Wilcoxon signed-rank test based on TOST between the ranges −3, +3 HU, −5, +5 HU, and −10, +10 HU.

	Corticomedullar Phase				Nephrographic Phase			
	*p* Value			CI 95%	*p* Value			CI 95%
Range (HU)	±3	±5	±10		±3	±5	±10	
All lesions	0.003	4.68 × 10^−9^	8.42 × 10^−15^	[0.8, 2.4]	0.002	2.19 × 10^−8^	2.03 × 10^−15^	[1, 2.54]
Renal masses	0.001	4.54 × 10^−5^	6.27 × 10^−6^	[−1.3, 1.5]	0.001	2.63 × 10^−5^	1.5 × 10^−6^	[−0.8, 2]
Cystic lesions	0.16 *	2.38 × 10^−5^	1.61 × 10^−10^	[1.4, 3.25]	0.07 *	6.26 × 10^−5^	2.47 × 10^−10^	[1.4, 3.3]

* Values with asterisk (*) do not show statistical significance (*p* value > 0.05). CI—Confidence Interval.

## Data Availability

Not applicable.

## Abbreviations

dl-DECT	dual layer-Dual Energy CT
HU	Hounsfield Unit
ROI	Region of Interest
SBI	Spectral Basis Images
TNC	True Non-Contrast
VNC	Virtual Non-Contrast
